# Turmeric Is Therapeutic *in Vivo* on Patient-Derived Colorectal Cancer Xenografts: Inhibition of Growth, Metastasis, and Tumor Recurrence

**DOI:** 10.3389/fonc.2020.574827

**Published:** 2021-01-19

**Authors:** Mingyue Li, Grace Gar-Lee Yue, Lianxiang Luo, Stephen Kwok-Wing Tsui, Kwok-Pui Fung, Simon Siu-Man Ng, Clara Bik-San Lau

**Affiliations:** ^1^ School of Biomedical Sciences, The Chinese University of Hong Kong, Hong Kong, China; ^2^ Institute of Chinese Medicine and State Key Laboratory of Research on Bioactivities and Clinical Applications of Medicinal Plants, The Chinese University of Hong Kong, Hong Kong, China; ^3^ The Marine Biomedical Research Institute, Guangdong Medical University, Zhanjiang, China; ^4^ Department of Surgery, Faculty of Medicine, The Chinese University of Hong Kong, Hong Kong, China

**Keywords:** colorectal cancer, patient-derived xenografts, turmeric, tumor recurrence, herbal medicines, network pharmacology

## Abstract

Colorectal cancer is the third most frequently diagnosed cancer worldwide. Clinically, chemotherapeutic agents such as FOLFOX are the mainstay of colorectal cancer treatment. However, the side effects including toxicity of FOLFOX stimulated the enthusiasm for developing adjuvants, which exhibit better safety profile. Turmeric extract (TE), which has been previously shown to suppress the growth of human and murine colon xenografts, was further demonstrated here for its inhibitory effects on colon cancer patient-derived xenografts (PDX). PDX models were successfully established from tissues of colon cancer patients and the PDX preserved the heterogeneous architecture through passages. NOD/SCID mice bearing PDX were treated either with TE or FOLFOX and differential responses toward these treatments were observed. The growth of PDX, metastasis and tumor recurrence in PDX-bearing mice were suppressed after TE treatments with 60% anti-tumor response rate and 83.3% anti-metastasis rate. Mechanistic studies showed that TE reduced tumor cell proliferation, induced cell apoptosis, inhibited metastasis *via* modulating multiple targets, such as molecules involved in Wnt and Src pathways, EMT and EGFR-related pathways. Nevertheless, FOLFOX treatments inhibited the PDX growth with sharp decreases of mice body weight and only mild anti-metastasis activities were observed. Furthermore, in order to have a better understanding of the underlying mechanisms, network pharmacology was utilized to predict potential targets and mechanism. In conclusion, the present study demonstrated for the first time that oral TE treatment was effective to suppress the growth of colon PDX and the recurrence of colon tumors in mice. The findings obtained from this clinically relevant PDX model would certainly provide valuable information for the potential clinical use of TE in colorectal cancer patients. The application of PDX model was well illustrated here as a good platform to verify the efficacy of multi-targeted herbal extracts.

## Introduction

Colorectal cancer (CRC) is the third most frequently diagnosed malignancy and is the second leading cause of cancer death worldwide (1). The incidence and death rates of CRC decreased among individuals aged ≥ 50 years, but increased by 13% in those aged less than 50 years (2). Metastasis results in nearly 90% of all cancer deaths (3) and it occurs in 20%–30% of CRC patients (2, 4). Poor prognosis of patients with non-resectable stage III–IV metastatic CRC (mCRC) provoked the development of therapeutics with novel mechanisms of action. Nonetheless, adverse events were reported in some targeted therapies (5, 6). Hence, there remains high unmet needs for safer adjuvant and/or therapeutic agents for mCRC patients, which could be searched from natural sources.

In the progress of development of anti-tumor and anti-metastatic agents, preclinical models play vital roles in translating preclinical data to clinical efficacy. However, high failure ratio of novel anti-tumor therapies in clinical trials partly results from the inability of conventional cell line xenograft models in reliably predicting the clinical efficacy (7, 8). In fact, cell lines used in xenograft models have adapted to the passaging on plastic outside of a natural tumor environment, whereas cancer cell-stromal cell interactions should have taken place in the presence of human extracellular matrix component. Hence, the cell line xenograft models may not be accurate enough to reflect interaction with the tumor microenvironment and tumor heterogeneity (8). In contrast, patient-derived xenograft (PDX) models, which have been established and adapted in recent years, are shown to preserve the intratumoral cell heterogeneity, histopathological and genetic alterations (9, 10). The PDX models are now regarded as effective, reproducible and clinically relevant preclinical models for testing the efficacy of new target-directed therapies, validating biomarkers of drug response, and evaluating preclinical drugs or therapeutic agents (11–13).

Along this line, natural product curcumin has been proven to have multiple molecular targets in cancer (14), and other components such as turmerones and polysaccharides have also been shown to possess anti-proliferative and immune-stimulatory effects (15, 16). However, poor bioavailability and low solubility limit curcumin to achieve its satisfactory therapeutic outcome in clinical trials. Previous studies suggested that turmeric (main ingredient in curry and a source of curcumin) may contribute to the lower CRC incidence in Indians (17, 18). Our previous studies also demonstrated that the whole complex of turmeric extract (TE) rather than curcumin alone could exert better inhibitory effects on colon cancer cell line generated xenografts growth in mice (19). Xenografts derived from cell lines generally exhibit poorly differentiated carcinomas that lack resemblance to the original patient tumor in terms of molecular characteristics (20). In order to confirm the benefits arising from the use of turmeric in clinical-relevant animal models, which would be a crucial step before launching clinical trial for this health supplement, we established a panel of PDX of CRC. The efficacies of TE on tumor growth, metastasis and tumor recurrence were then investigated in this panel of PDX for the first time.

Systems biology approach, in particular network pharmacology enable the paradigm shift from “one-target, one-drug” to a “network-target, multiple-component-therapeutics” mode, which is a powerful tool for the analysis of drug combinations, especially for traditional Chinese medicines (21, 22). Network pharmacology is a new method based on “disease-gene-drug” network which provides a new way to explore the mechanism of drug action at molecular, cellular, tissue and biological levels (23). The multi-dimensional mechanism of drug action is evaluated by target prediction, pharmacokinetic measurement and network analysis. More importantly, network pharmacology has been successfully applied to predict/identify the molecular mechanisms of traditional Chinese medicine in the treatment of various diseases (21), including cardiovascular diseases (24), neurodegenerative diseases (25), cancer (26), etc. Here, we have identified potential active components of TE against CRC targets, and predicted their network, suggested the potential pathways and key regulatory genes.

## Method and Materials

### Reagents

Turmeric ethanolic extract (TE) was prepared as previously reported (19, 27). In brief, the dried rhizome of *Curcuma longa* was extracted under reflux using 95% ethanol and evaporated under reduced pressure at 60°C to dryness. Quantification of two commercially available chemical markers, curcumin and Ar-turmerone (Sigma-Aldrich, MO, USA), in turmeric ethanolic extract was performed using by UPLC and the chemical profiles were registered (27). The contents of curcumin and Ar-turmerone present in turmeric ethanolic extract were 18.7% (w/w) and 5.3% (w/w), respectively. Standardization of turmeric ethanolic extract used in the present study was same as the method reported previously, in which the 3-D chromatograms of the extract as well as the extracted ion chromatograms (EIC) and MS spectra of the marker compounds were also well reported previously (27).

Chemotherapeutics FOLFOX consisted of fluorouracil, folinic acid and oxaliplatin, were obtained from Sigma-Aldrich (MO, USA). Primary antibodies against RhoA, β-catenin, Src, p-Src, and GAPDH were purchased from Cell Signaling (MA, USA); Ki-67 from Abcam (MA, USA); CD31 from Dako (CA, USA); mouse/rabbit specific HRP/DAB (ABC) Detection IHC kit from Abcam Inc (Cambridge, UK). The *in situ* Cell Death Detection kit for TUNEL staining was from Roche (IN, USA). The Trizol reagent was from Thermo Fisher Scientific (MA, USA) and QuantiFast^®^ SYBR^®^ Green PCR mini kit was from Qiagen (CA, USA).

### Establishment of Colorectal Cancer Patient Derived Xenograft Mouse Model

Experimental protocols and procedures were reviewed and approved by the Joint Chinese University of Hong Kong – New Territories East Cluster Clinical Research Ethics Committee (CREC Ref No.: 2016.444) and the Animal Experimentation Ethics Committee of The Chinese University of Hong Kong (Ref No. 15-164-MIS).

**Figure 1:**
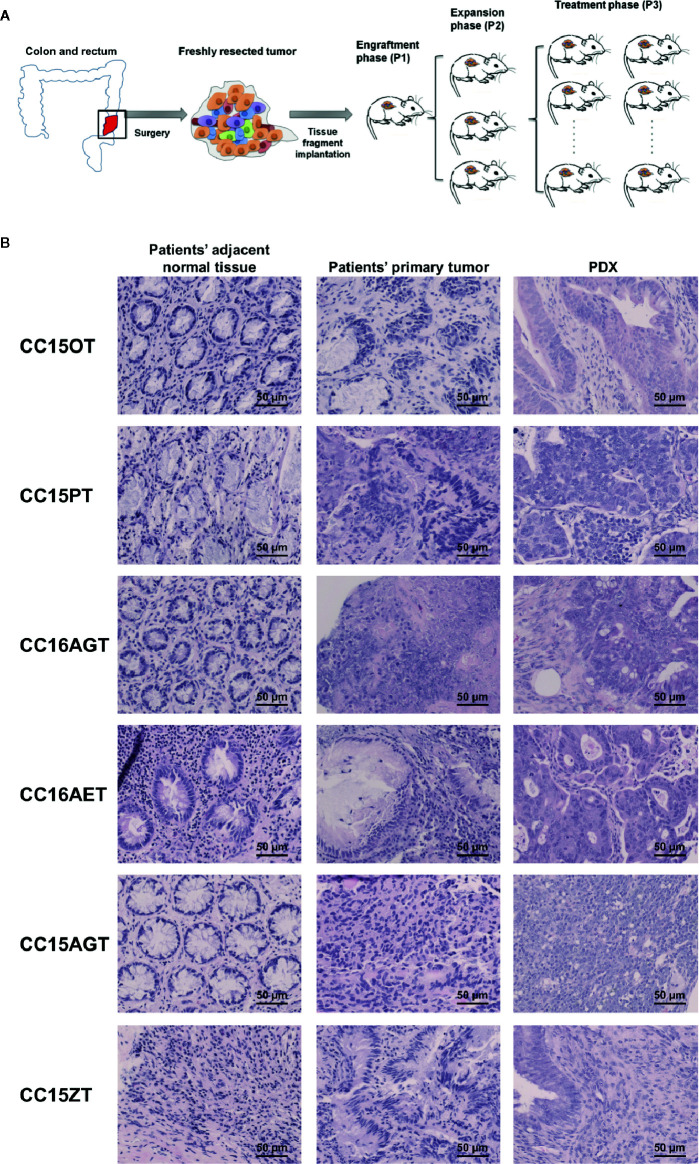
Schematic diagram showing the establishment of PDX samples and histological assessment of PDX samples. **(A)** Patients’ tumors were obtained, inoculated subcutaneously on the back of NOD-SCID mice (P1). When the size of PDX samples reached 1,000 mm^3^, PDX samples were sub-cultured into the next batch of mice (P2), which subsequently expanded to the third passage (P3). Treatments of turmeric extract or FOLFOX or vehicle control were applied in the mice bearing P3 PDX samples. **(B)** The histology of representative patients’ adjacent normal tissues, patients’ primary tumors and PDX were determined using H&E staining. Bar = 50 μm.

Freshly resected tissues (tumor and adjacent normal tissues) from consented CRC patients were collected after colon surgery. Half portion of resected tumor tissues were stored in −80°C freezer for further histological and molecular analysis and another half portion of tumors were transferred at 4°C into RPMI-1640 medium with antibiotics. Within 24 h of surgical resection, tumor tissues were trimmed, cut into 3- to 5-mm sizes and inoculated subcutaneously on the back of 6- to 8-week-old NOD/SCID mice, which were supplied by the Laboratory Animal Services Centre of The Chinese University of Hong Kong. Mice were maintained in pathogen-free conditions in specifically designed air-controlled rooms with a 12-h light/dark cycle. When the tumors reached 1,000–1,500 mm^3^, mice with the first generation of xenografts (P1) were sacrificed and then the xenografts were isolated and expanded for two more generations (P2 and P3). When P3 xenografts reached an average volume of 50 mm^3^, mice were then subjected to TE or FOLFOX treatments (**Figure 1A**).

### Administration of Turmeric Extract or FOLFOX to PDX-Bearing Mice

In total, seven batches of PDX samples were established for the following pharmacological experiments. Six batches of PDX-bearing mice were treated with TE (200 mg/kg, orally administered) or vehicle control 6 days a week for 2–8 weeks depending on the tumor sizes and the well-being of mice. FOLFOX (15 mg/kg fluorouracil and 5 mg/kg folinic acid daily plus 5 mg/kg oxaliplatin once a week) was administered intraperitoneally for 2 or 4 weeks depending on the well-being of mice. The treatments and assessment details of the PDX samples were listed in **Table 1**. In our previous study, TE at 400 mg/kg has been shown to inhibit growth of human colon HT29 subcutaneous xenografts (27). Thus, both 200 and 400 mg/kg TE have been tested in our pilot studies in PDX-bearing mice. Results showed that the inhibitory activities of these two doses of TE were comparable and without significant difference. Hence, the minimum effective dose (200 mg/kg) was chosen for the subsequent PDX experiments in the present study.

**Table 1:** Treatment and assessment details of PDX-bearing mice.

(A) Treatment and assessment details of TE-treated PDX-bearing mice.							
PDX samples	Treatments	Duration		Tumor collection after treatments		Tumor size changes shown in **Figure 2**	Lung and liver metastasis assessment
**CC15OT**	TE or vehicle control	8 weeks		Yes		Yes	Yes
**CC15PT**	TE or vehicle control	2 weeks		Yes		Yes	Yes
**CC16AGT**	TE or vehicle control	5 weeks		Yes		Yes	Yes
**CC16AET**	TE or vehicle control	4 weeks		Yes		No^a^	Yes
**(B) Treatment and assessment details of FOLFOX-treated PDX-bearing mice.**							
**PDX samples**	**Treatments**		**Duration**	**Tumor collection after treatments**		**Tumor size changes shown in Figure 7**	**Lung and liver metastasis assessment**
**CC16AGT**	FOLFOX or vehicle control		2 weeks	Yes		Yes	Yes
**CC15AGT**	FOLFOX or vehicle control		4 weeks	Yes		Yes	Yes
**(C) Treatment and assessment details of TE-treated PDX-bearing mice with surgical removal of original PDX.**							
**PDX samples**	**Treatments**	**Duration**		**Tumor size changes shown in Figure 6**	**Recurrent tumors collection**		**Lung and liver metastasis assessment**
**CC15ZT**	TE or vehicle control	7 weeks		Yes	Yes, 4 weeks after surgery		Yes
**CC16ANT**	TE or vehicle control	4 weeks		Yes	Yes, 5 weeks after surgery		Yes

a Tumor sizes of TE-treated and vehicle control groups were not significantly different so that the results have been shown in **Supplementary Information Fig. S4**.

Tumor volumes and body weights were measured once a week until the end of experiments. Tumor volumes were calculated using the formula: length × width × depth/2 (mm^3^) as described previously (28). Tumor growth inhibition was calculated using the formula: (TGI= Average tumor weight in control group − Average tumor weight in treatment group)/Average tumor weight in control group × 100%. Relative tumor volume was equaled to tumor volume (on the measuring day) divided by tumor volume (on day 0).

### Histological Assessments

PDX samples were collected at the end of experiments and separated into two halves, one half was snap frozen at −80°C for further Western blot/RT-PCR analysis and the other half was fixed in 10% formalin. Lungs and livers were also fixed in 10% formalin, embedded in paraffin and then sectioned for hematoxylin and eosin (H&E) staining. Tumor area in liver and lung H&E sections were used to assess the level of metastasis. The metastatic area was calculated as percentage of metastatic area in whole view of photo. Embedded PDX tissues from each group were sectioned and stained with H&E or subjected to IHC staining against β-catenin and Ki 67 antibodies. The TUNEL assay was performed to assess apoptosis in tumor sections as per kit’s instruction. H&E and IHC staining were performed as previously described (28). Immunoreactivity was analyzed in 5 random areas for each tumor tissue and was scored as 0 (no staining), 1 (weak staining), 2 (moderate staining), 3 (strong staining), and 4 (very strong staining) (29).

### Western Blotting

Western blot was performed using the lysates of PDX samples. Twenty to sixty micrograms of protein were loaded to denaturing gel electrophoresis. After transfer to PVDF membrane, membranes were blocked with 5% BSA and incubated overnight with primary antibodies, RhoA, p-Src, Src, β-catenin, cleaved caspase-3, and GAPDH in 1:1,000 dilutions. Blots were then incubated with secondary antibodies (1:2,000). Quantification was performed using Image J software as described previously (28).

### RNA Extraction and RT-PCR

Total RNA was extracted from three batches (CC15OT, CC15PT, and CC15ZT) of PDX samples using Trizol reagent. The reverse transcription and quantification were performed as previously described (30). The gene expression of Wnt signaling elements (AXIN2, DKK1, and SMAD7), EMT pathway related molecules (N-cadherin, Snail and Vimentin, and EpCAM), growth factor receptors [platelet-derived growth factor receptor B (PDGFRB), epidermal growth factor receptor (EGFR)], asparagine synthetase (ASNS), Rho signaling (RTKN2), ATP-binding cassette transporters (ABCA13), and iNOS were evaluated using qPCR. Besides, total RNA of CC15OT primary tumor and adjacent normal tissue samples were extracted and subjected to qPCR for the expressions of KRAS and EGFR. In addition, total RNA of lungs CC16AET were extracted and the expressions of human-specific 850-bp fragment of the α-satellite DNA on human chromosome 17 (Ch17) were detected by qPCR (31, 32). In brief, real time semi-quantitative PCR of cDNA samples were performed in Bio-Rad CFX96™ Real-time system C1000 Thermal cycler using the QuantiFast SYBR Green RT-PCR kit (Qiagen, USA). The primers sequences were listed in **Table S1** (in supplementary information), which were synthesized by Thermo Fisher Scientific (Hong Kong). The specific gene mRNA levels (including Ch17 expression level) were normalized to GAPDH and expressed as a fold change compared to the vehicle control group.

### Data Preparation and Analysis of Network Pharmacology

The potential active compounds and putative targets of turmeric were searched using Traditional Chinese Medicine Systems Pharmacology Database (TCMSP, http://tcmspw.com/tcmsp.php), and The Encyclopedia of Traditional Chinese Medicine (ETCM, http://www.tcmip.cn/ETCM/index.php/Home/Index/index.html). The optimal cutoff values of OB and DL in TCMSP were set to 30% and 0.18. Information on associated target genes of CRC was obtained from GeneCards (https://www.genecards.org/), and the top 1,000 genes were reserved for further analysis. The String database (http://string-db.org/) was utilized to obtain the data of protein-protein interaction (PPI) with the species limited to “Homo sapiens”. The Cytoscape 3.7.2 software was applied for constructing the Compound-target Network, Compound-candidate target Network, PPI Network, and Hub Genes Network. GO Slim of candidate targets was analyzed by WebGestalt (http://www.webgestalt.org/), and the biological processes and KEGG pathway were analyzed by DAVID 6.8 (https://david.ncifcrf.gov/).

### Statistical Analysis

All quantitative data were expressed as mean ± standard error of the mean (SEM). The unpaired Student’s *t*-test was conducted to determine statistical significant differences. *p <* 0.05 was considered as statistically significance.

## Results

### Generation of PDX From Colon Cancer Patients’ Tumor Samples

PDX samples for colon cancer were established from seven patients’ tumor samples. PDX were successfully grown through three serial passages in mice. The latency time from initial inoculation time to post-treatment varied from 6 to 8 months. Clinical characteristics including tumor size, origin of samples, tumor/node/metastasis stage and survival time, etc. were listed in **Table 2**. All patients’ tumors included in this study were moderately differentiated without metastasis. Histological examination of the H&E stained PDX sections was conducted to compare the similarity of PDX with the original patients’ tumors. Both the primary tumors and PDX show features of adenocarcinoma (**Figure 1B**). PDX could represent diverse clinical characteristics typically observed in patients according to pathologist’s assessment. Meanwhile, the adjacent normal tissues were also stained by H&E to illustrate the differences between normal tissues and tumor tissues. Most of the adjacent tissues have well differentiated histomorphology, which were apparently different from tumor tissues.

**Table 2:** Characteristics of the patients for CRC PDX model.

Sample codes	CC15OT	CC15PT	CC16AGT	CC15AGT	CC15ZT	CC16ANT	CC16AET
Range of age	70-74	80-84	50-54	65-69	80-84	65-69	60-64
Metastases at presentation (Y/N)	N	N	N	N	N	N	N
Tumor length (cm)	2.5	4	6	3.5	9	4	6
Origin of sample	sigmoid	rectum	ascending colon	rectum	ascending colon	splenic flexure	transverse colon
Number of lymph nodes removed	23	13	43	32	20	29	31
Tumor/node (TN) staging	T3N0	T2N0	T3N0	T3N0	T2N0	T3N0	T3N0
Tumor differentiation (well/moderate/poor)	moderate	moderate	moderate	moderate	moderate	moderate	moderate
Lymphovascular permeation (Y/N)	N	N	N	N	N	N	N
Adjuvant chemotherapy (Y/N)	N	N	N	N	N	N	N
Adjuvant radiotherapy (Y/N)	N	N	N	N	N	N	N
Follow up time from surgery (months)	42	41	30	35	37	23	32
Recurrence (Y/N)	N	N	N	N	N	N	N

### Turmeric Extract Inhibited Tumor Growth in Colon PDX

**Figure 2:**
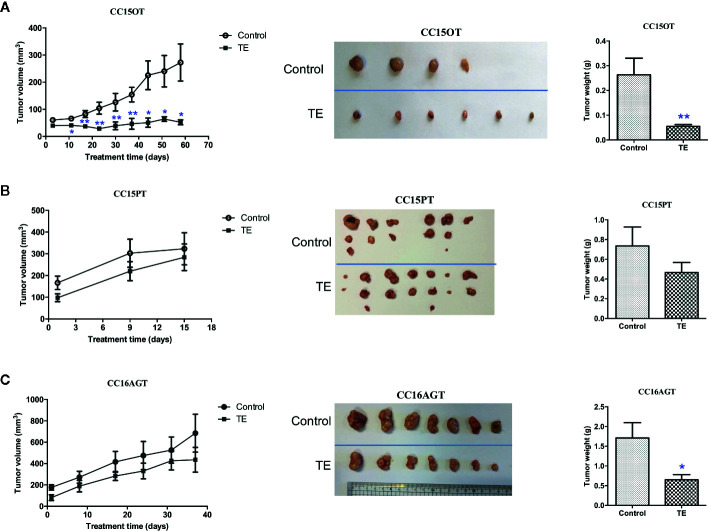
Effects of TE treatment in 3 PDX samples CC15OT, CC15PT, and CC16AGT. The tumor volume and weight in the control group and TE treatment groups were compared using **(A)** CC15OT (n = 4–6), **(B)** CC15PT (n = 6–7), and **(C)** CC16AGT (n = 7). Tumor volume was measured once a week while the final tumor weight was measured at the end of the experiment. Representative photos for tumors from 4 to 7 mice in each PDX sample. Data were presented as mean ± SEM, ^*^ *p* < 0.05, ^**^ *p* < 0.01 vs. vehicle control at the same time point.

Mice bearing 4 batches of PDX samples (CC15OT, CC15PT, CC16AGT, and CC16AET) were received TE treatments. Since the size of the original patients’ tumors varied, the number of mice in each batch of PDX samples were different. Besides, the duration of treatment was determined by the tumor size and/or the well-being of mice. Some of the experiments were terminated when the tumor size of untreated control group was large (~600–700 mm^3^) or the PDX-bearing mice became very weak (even the PDX size was not large), as it was unethical to continue the experiements. Nevertheless, our results could show that oral TE treatment decreased the size of PDX in three batches (except CC16AET) with different extent. As shown in **Figure 2**, all tumors in TE-treated groups were smaller than those in vehicle control groups, with tumor growth inhibition for CC15OT, CC15PT, and CC16AGT at 79.1%, 36.7%, and 51.5%, respectively. The inhibitory effects of TE were shown to be the most effective in CC15OT sample, the final tumor weight of TE-treated group was 54.9 ± 7.43 g versus that of control group 263.2 ± 67.3 g (*p* < 0.01, **Figure 2A**). Body weights of mice did not significantly differ between treatment and control groups in these three tested batches, indicating TE did not cause systemic toxicity in mice.

### Anti-Metastatic Effect of TE Treatment in Colon PDX Models

**Figure 3:**
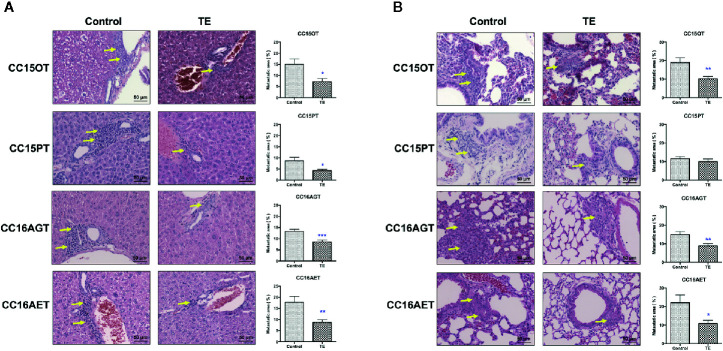
Effects of TE treatment on liver and lung metastasis of PDX samples. **(A)** Livers and **(B)** lungs of mice bearing CC15OT, CC15PT, CC16AGT, and CC16AET PDX samples were collected and subjected to histological analysis. Liver and lung sections were stained with H&E and the metastatic area (shown by yellow arrows) was assessed. Representative photos for 4-8 livers or lungs in each group. Quantitative results of liver and lung metastasis were shown on the right panel. Data shown were mean ± SEM. ^*^ *p* < 0.05, ^**^ *p* < 0.01, ^***^ *p* < 0.001 vs. vehicle control.

The efficacy of TE treatment on tumor metastasis in PDX models was reflected by metastasis area in livers and lungs. Levels of metastasis varied among batches, metastatic area ranged from 7.8% to 20.9% in lungs, and from 6.3% to 17.7% in livers. The highest tumor burden was observed in CC16AET sample, with the metastatic area as 17.7% and 20.9% in liver and lung, respectively. The liver metastasis in four batches of PDX samples (CC15OT, CC15PT, CC16AGT, and CC16AET) could be significantly decreased after TE treatment (**Figure 3A**).Whereas TE treatment significantly reduced the lung metastatic area in CC15OT, CC16AGT, and CC16AET samples (**Figure 3B**). Importantly, most of the metastasis occurred near blood vessels where the tumor cells permeated from and then colonized. As the highest inhibition rate (51.0%) of lung metastasis by TE treatment was observed in CC16AET sample, the expression of the human-specific 850-bp fragment of the α-satellite DNA on human Ch17 (31, 32) was determined in lung tissues. Results showed that the relative Ch17 expression (after normalization with housekeeping gene GAPDH) in TE-treated group was 1.04 ± 0.70, while that in control group was 2.80 ± 1.56. There was significantly decreased of the Ch17 expression in TE-treated mice (*p* < 0.05), suggesting that the inhibitory effect of TE treatment on the metastasis and/or colonization of human xenograft cells in mice lungs.

### Underlying Mechanisms of Anti-Tumor and Anti-Metastatic Effects of TE

**Figure 4:**
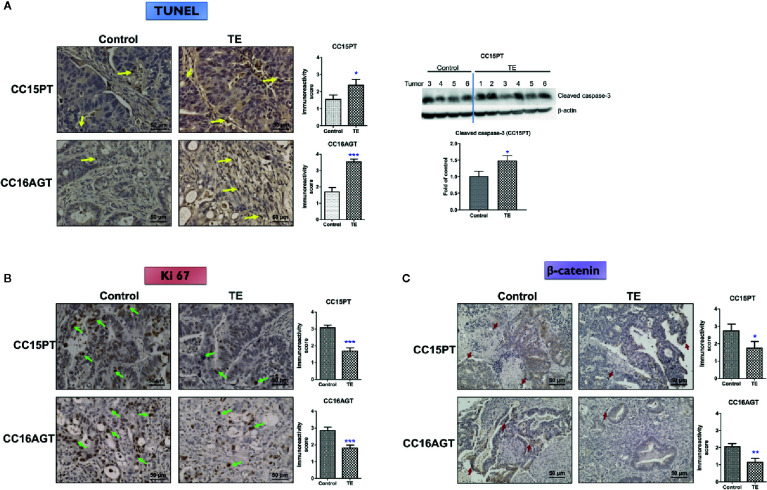
Mechanism of the inhibitory effects of TE treatment on PDX samples. Representative images of **(A)** TUNEL, IHC staining against **(B)** Ki 67, and **(C)** β-catenin in tumor sections from two batches (CC15PT and CC16AGT) of PDX-bearing mice. Bar = 50 μm. Quantitative results of positive-stained cells in the tumor sections were shown on the right panel. Western blots of cleaved caspase-3 in CC15PT were shown in **(A)**. Data were presented as mean ± SEM. n = 20–25 sections in each group. ^*^ *p* < 0.05, ^**^ *p* < 0.01, ^***^ *p* < 0.001 vs. vehicle control.

**Figure 5:**
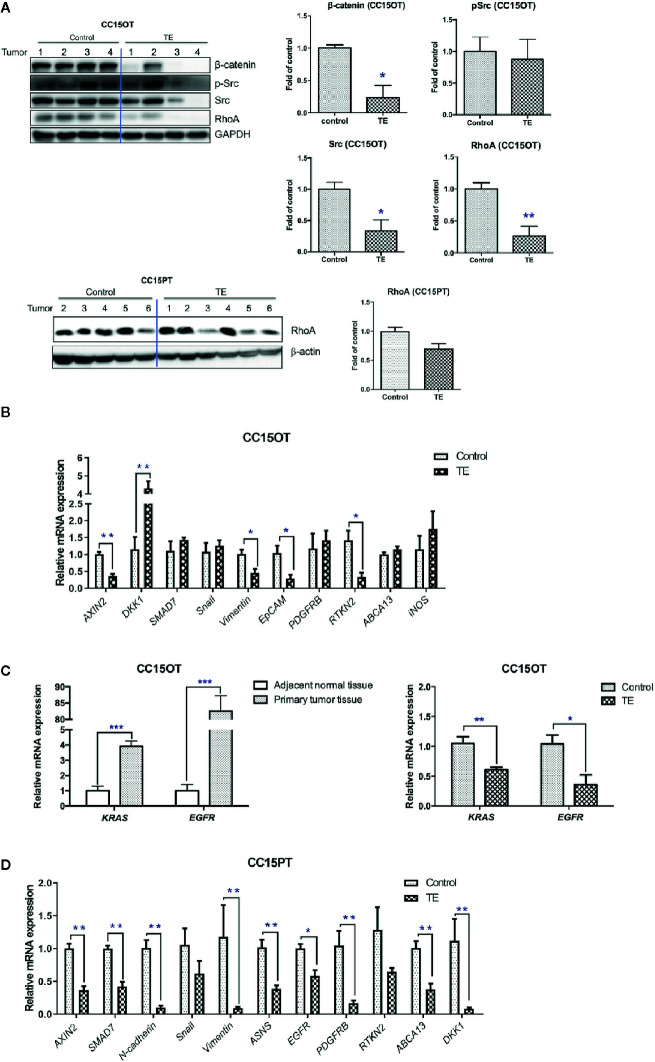
Modulatory effects of TE treatment on protein and gene expressions in PDX samples. **(A)** Western blot of tumor protein against β-catenin, p-Src/Src, RhoA, GAPDH in CC15OT, and CC15PT PDX samples. The quantitative data presented as fold of vehicle control. **(B–D)** Real-time PCR of selected gene expressions in CC15OT and CC15PT PDX samples. GAPDH was used for normalization. n = 4–6 tumors in each groups. Data were shown as mean ± SEM. ^*^ *p* < 0.05, ^**^ *p* < 0.01, ***p < 0.001 vs. vehicle control.

As the tumor sizes were reduced after TE treatment, the tumor sections were subjected to TUNEL assay for evaluating apoptosis in tumors. As shown in **Figure 4A**, TE treatment could significantly induce apoptosis in tumors in CC15PT and CC16AGT samples as shown by TUNEL asssy and the expression of cleaved caspase-3. Besides, TE treatment decreased the expression of Ki 67 (proliferation marker, **Figure 4B**) in tumor sections. On the other hand, β-catenin, involving in tumor cell proliferation (33) and EMT process (34) which is considered as the first step in tumor metastasis, was also assessed in tumor sections. In CC15PT and CC16AGT samples (**Figure 4C**), β-catenin expression was shown to be significantly decreased in tumors. As the sizes of TE-treated tumors in CC15OT batch were small, tumors were subjected to the extraction of proteins and RNA instead of immunohistochemical analysis. The protein expression of β-catenin in CC15OT samples was examined using Western blot as shown in **Figure 5A**, and it was apparently reduced after TE treatment.

Furthermore, TE treatment significantly reduced the protein expressions of β-catenin, Src and RhoA in CC15OT (*p* < 0.05) and slightly reduced the protein expressions of RhoA in CC15PT samples (*p* = 0.074, **Figure 5A**). As the RNA of CC15OT and CC15PT samples were extracted, the gene expression of Wnt signaling elements (*AXIN2*, *DKK1*, and *SMAD7*), EMT pathway related molecules (N-cadherin, Snail and Vimentin, and EpCAM), growth factor receptors (*PDGFRB*), asparagine synthetase (*ASNS*), Rho signaling (*RTKN2*), ATP-binding cassette transporters (*ABCA13*), and *iNOS* were evaluated using qPCR. As shown in **Figure 5B**, mRNA expressions of Wnt signaling elements (*AXIN 2*), EMT pathway related molecules (Vimentin and EpCAM), *EGFR*, and *RTKN2* were all down-regulated in TE-treated tumors of CC15OT samples, whereas *DKK1* was significantly up-regulated by TE treatment. On the other hand, the expressions of *KRAS* and *EGFR* in primary tumor tissues were shown to be significantly higher than those in adjacent normal tissues (*p* < 0.001, **Figure 5C**, left panel). While the CC15OT PDX-bearing mice treated with TE, the expressions of these two genes in the PDX samples were significantly decreased when compared with untreated control (*p* < 0.05, **Figure 5C**, right panel).

Furthermore, in the samples of CC15PT, the expressions of *AXIN2*, *SMAD7*, N-cadherin, vimentin, *ASNS*, *EGFR*, *PDGFRB*, *ABCA13*, and *DKK1* were all significantly down-regulated after TE treatment (**Figure 5D**). These results suggested the multi-targeted activities of TE treatment on the colon PDX.

### Novel Finding of TE in Reducing Colon Tumor Recurrence

**Figure 6:**
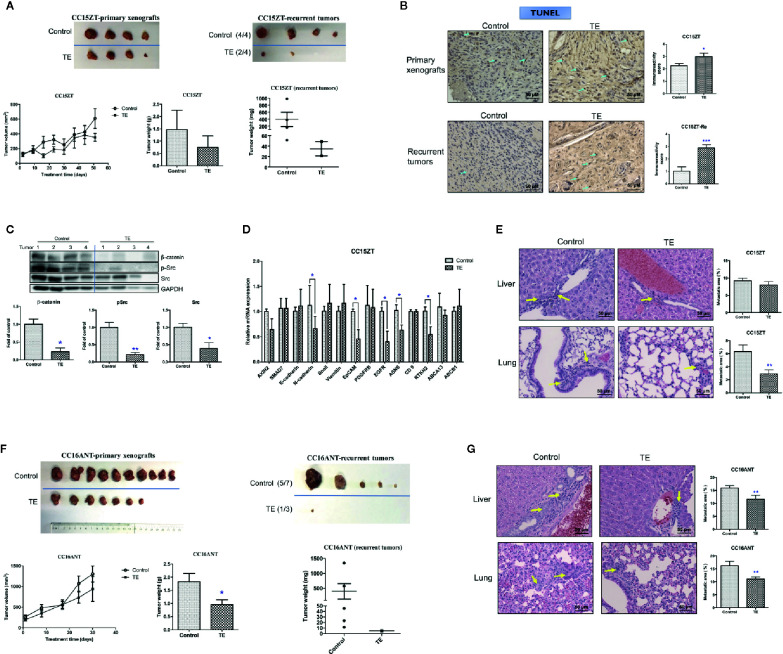
Effects of TE treatment on the growth of PDX and the recurrence of tumor. Mice bearing PDX samples CC15ZT and CC16ANT were treated with TE and the primary xenografts were removed by surgery. For the CC15ZT PDX sample, **(A)** tumor volume changes, final tumor weight and photo of primary xenografts were shown on the left panel. The final tumor weight and photo of recurrent tumors were shown on the right panel. The tumor incidence was listed in the brackets next to the photo. **(B)** Representative images of TUNEL in primary xenografts and recurrent tumor sections and quantitative results of positive-stained cells in the tumor sections were shown. Bar = 50 μm. Data were presented as mean ± SEM. n = 20 sections in each group. **(C)** Western blot of primary xenograft protein against β-catenin, p-Src, Src, and GAPDH and the quantitative data presented as fold of vehicle control. **(D)** Real-time PCR of selected gene expressions in the primary xenografts. GAPDH was used for normalization. n = 4 tumors in each group. **(E)** Liver and lung sections were stained with H&E and the metastatic area (shown by yellow arrows) was assessed. n = 20 sections in each group. Quantitative results of liver and lung metastasis were shown on the right panel. For the CC16ANT PDX sample, **(F)** tumor volume changes, final tumor weight and photo of primary xenografts were shown on the left panel. The final tumor weight and photo of recurrent tumors were shown on the right panel. The tumor incidence was listed in the brackets next to the photo. **(G)** Liver and lung sections were stained with H&E and the metastatic area (shown by yellow arrows) was assessed. n = 15–25 sections in each group. Data were shown as mean ± SEM. ^*^ *p* < 0.05, ^**^ *p* < 0.01, ^***^ *p* < 0.001 vs. vehicle control.

Since the anti-tumor and anti-metastatic efficacies were observed in PDX-bearing mice treated with TE, the potential inhibitory effect of TE treatment on tumor recurrence was further investigated in another 2 batches of PDX samples. Mice bearing PDX samples CC15ZT and CC16ANT were treated with TE for 7 and 4 weeks, respectively. Then, the xenografts were removed by surgery. The primary tumor volumes of TE-treated groups were smaller than control group, and the average tumor weight in control group was 1.47 ± 0.39 g versus 0.74 ± 0.23 g in TE-treated group (**Figure 6A**). After the mice recovered from surgery, they were maintained without any treatment in the following 4 weeks. The recurrent tumors could be observed in mice of vehicle control group. The mice were sacrificed when the largest recurrent tumors reached the size of 1,000 mm^3^, and the recurrent tumors, livers and lungs were collected for analysis. The tumor incidence after surgery and recurrent tumor weight could indicate the inhibitory effect of TE on tumor recurrence. The incidence of tumor in vehicle control group was 100% (4/4), while that in TE treatment group was only 50% (2/4) (**Figure 6A**). The average weight of recurrent tumors in TE treatment group (0.018 ± 0.012 g) were lower than that in control group (0.393 ± 0.207 g). TUNEL assay for the primary xenografts and recurrent tumors revealed that TE treatment induced apoptosis in tumors (**Figure 6B**). The protein expressions of β-catenin, Src and pSrc were suppressed in the tumors in TE-treated group (**Figure 6C**). The gene expressions of EMT pathway related molecules (N-cadherin, EpCAM), *EGFR*, *ASNS*, and *RTKN2* were also down-regulated after TE-treatment (**Figure 6D**). All these molecular alterations in the primary xenografts might contribute to the inhibitory effects of TE on the growth of primary and recurrent tumors. Lastly, lung metastasis was found to be significantly reduced after TE treatment (**Figure 6E**).

The inhibitory effect of TE on tumor recurrence was also demonstrated in mice bearing PDX sample CC16ANT, which were treated with TE for 4 weeks, and the primary xenografts were removed by surgery. The average tumor weight in vehicle control group was 1.82 ± 0.32 g while that in TE-treated group was 0.95 ± 0.19 g, which was significantly decreased (**Figure 6F**, left panel). Five weeks after the removal of the primary xenografts, recurrent tumor was found in 1 out of 3 mice treated with TE (i.e., tumor incidence: 1/3). While in vehicle control group, five mice out of seven were found to have recurrent tumors (**Figure 6F**, right panel). Similarly, the significant reduction of metastasis in liver and lung could be observed in this set of experiment (**Figure 6G**).

### Efficacy of FOLFOX in Colon PDX Model

**Figure 7:**
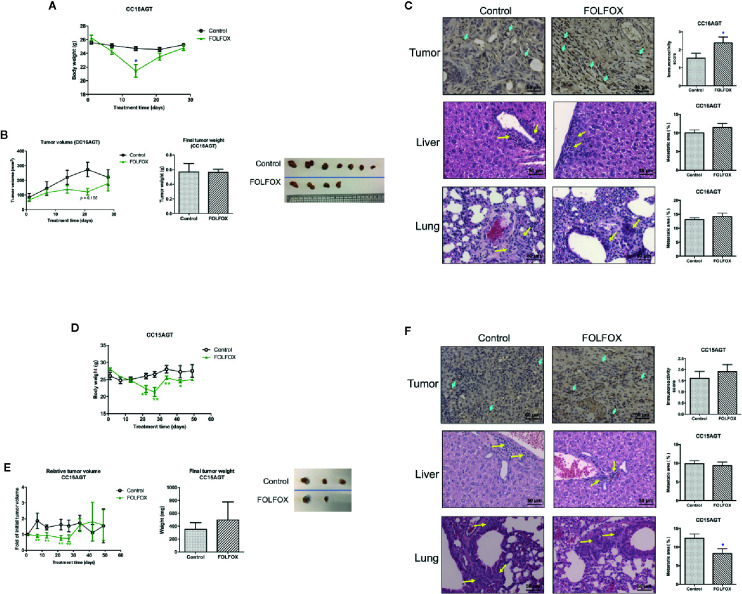
Effects of FOLFOX treatment on the tumor growth and metastasis in PDX samples. Mice bearing PDX samples CC16AGT and CC15AGT were treated with FOLFOX. **(A, D)** Body weight changes during treatment and final body weight. **(B, E)** Tumor volume changes/relative tumor volume changes, final tumor weight and photo of tumors. Data were presented as mean ± SEM. ^*^ *p* < 0.05, ^**^ *p* < 0.01 vs. vehicle control at the same time point. Representative images of TUNEL of tumor sections, H&E stained lung and liver sections of **(C)** CC16AGT samples and **(F)** CC15AGT samples. Quantitative results of positive-stained (TUNEL) cells in the tumor sections and the metastatic area were shown on the right panel. Bar = 50 μm. n = 10–35 sections in each group. Data were shown as mean ± SEM. ^*^ *p* < 0.05, ^**^ *p* < 0.01 vs. vehicle control.

Beyond surgery or in metastatic phase, FOLFIRI (fluorouracil, folinic acid, and irinotecan) or FOLFOX (plus oxaliplatin) are utilized as system treatment (35). Previous studies demonstrated the side effect of FOLFOX including neurotoxicity, immune suppression, hair loss, nausea, etc. (28, 36). Therefore, there is a strong desire for developing potent and safe anti-tumor and anti-metastatic therapeutics. In order to show the potential superior efficacy of TE over the conventional therapeutics, the efficacy of FOLFOX was also examined in the present study. Chemotherapeutics FOLFOX was administered to mice bearing CC16AGT and CC15AGT PDX samples. Surprisingly, the mice cannot even bear long time FOLFOX treatment. After 2 and 4 weeks of FOLFOX treatments, gradually decreases (>20%) of body weights (**Figures 7A, D** **)**, the treatments were observered and the treatments were terminated at 2 and 4 weeks in CC16AGT and CC15AGT PDX samples, respectively. The mice were maintained without any treatment until the end of experiments.

In the CC16AGT PDX samples, the tumor volume was smaller in FOLFOX treatment group when compared with vehicle control group (days 14–21). However, the final tumor volume and weight were similar in FOLFOX-treated group and the vehicle control group (**Figure 7B**), which might due to the termination of FOLFOX treatment on day 15. Nonetheless, the anti-tumor activities of FOLFOX could still sustain as the apoptotic area in tumors was higher in FOLFOX-treated group than that in vehicle control group (**Figure 7C**). The metastasis in liver and lung were also examined after FOLFOX treatment, and results showed that the metastatic area was slightly increased in FOLFOX-treated group (**Figure 7C**).

For the CC15AGT PDX samples, which should be sensitive to FOLFOX treatment because the tumor growth was suppressed by FOLFOX treatment as shown in the tumor growth curve (**Figure 7E**), meanwhile the body weight was decreased gradually (**Figure 7D**). After termination of FOLFOX treatment, the tumor volume of this group increased sharply (**Figure 7E**), resulting in comparable tumor size with vehicle control group. The apoptotic area of tumors were slightly higher in FOLFOX treatment group than that in vehicle control group (**Figure 7F**). Nevertheless, the metastasis in lung was found to be suppressed by FOLFOX treatment in this batch of PDX-bearing mice, further suggesting the diverse responses of PDX samples toward chemotherapeutics.

### Network Pharmacology Predicts Potential Mechanism of Turmeric Extract Treatment in Colorectal Cancer

**Figure 8:**
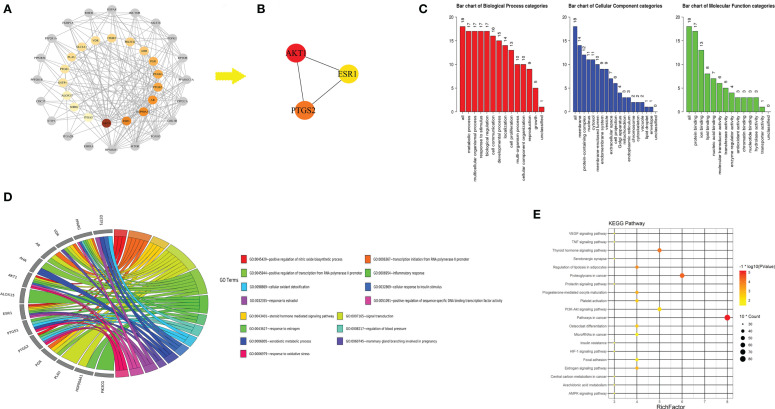
Protein-protein interaction (PPI) network of turmeric extract treated colorectal cancer. **(A)** Node representation of candidate target with greater degree was described by darker color. Nodes in grey was ranking neighbors of candidate targets. **(B)** Hub genes in candidate targets. **(C)** GO slim analysis of candidate targets from WebGestalt. **(D)** Biological process (BP) from DAVID 6.8 showing the top 15 BPs of turmeric extract against colorectal cancer. **(E)** KEGG analysis of candidate targets from DAVID 6.8.

A total of 18 candidate targets for treating CRC were collected from 14 potential active compounds (**Table S2**) of TE. The PPI network of candidate targets contained 38 nodes and 202 edges, in which average node degree is 10.6 (**Figure 8A**). The cytoHubba plugin of the Cytoscape software was used to analyze the 38 nodes and found that 3 targets belonged to the hub genes in candidate targets according to rank by Degree, Closeness, and Betweenness (**Figure 8B**). Hence, we suspected that the following three genes encode proteins in pivotal roles: AKT1, ESR1, and PTGS2. We then performed a GO Slim analysis by WebGestalt, and it was suggested that the candidate targets participated in protein binding, ion binding, and lipid binding in membrane, protein-containing complex, and nucleus (**Figure 8C**). To further examine the signaling pathways and functions of these candidate targets, functional enrichment analysis was carried out using DAVID 6.8. Candidate targets were found involved in biological processes such as signal transduction, inflammatory response, and positive regulation of nitric oxide biosynthetic process (**Figure 8D**). The results of KEGG analysis indicated that candidate targets were intensively associated with PI3K-Akt signaling pathway and thyroid hormone signaling pathway (**Figure 8E**). In fact, previous studies have shown that these signaling pathways are related to regulating cancer cell growth and proliferation (33, 34), which indicates a possible direction for further cancer related research. Except three hub genes and pathways in cancer, other targets of TE which could be potential targets in treating other diseases were also proposed.

## Discussion

The *in vivo* efficacies of turmeric ethanolic extract, which contains absorbable curcumin, were demonstrated in mice bearing colon PDX for the first time in the present study. Seven PDX samples were established from the moderately differentiated colorectal tumors from consented patients and the comprehensive evaluation on anti-tumor (including tumor recurrence) and anti-metastatic effects of TE and chemotherapeutics FOLFOX have been conducted. In our established PDX samples, histological features were well preserved through passages, despite the high degree of heterogeneous architecture and molecular characteristics, they could recapitulate *in vivo* tumor biology and be employed in the accurate preclinical drug evaluation (8). The responses of these PDX samples toward therapeutic agents (natural or synthetic) would be more clinically relevant than those from cell-derived xenograft models.

In view of the multiple entities of pathology of CRC, PDX model would be the most appropriate model to allow accurate testing of potential multi-targeted therapeutics. Recent studies demonstrated the use of combination of targeted drugs, e.g., ABT-263 plus YM-155 (37), panitumumab plus TAS-102 (38) in colon PDX models. In fact, apart from those novel synthetic small molecules and biomolecules (antibodies) for cancer treatment, herbal medicines are certainly alternative source of therapeutic agents since herbal medicines have long been used for chronic disorders management. The multi-components properties of herbal medicines would match the multiple combination treatment approaches in many diseases, such as cancer.

To prove this concept, a well-known Chinese and Ayurvedic herbal medicine, turmeric, the dried rhizome of the plant *Curcuma longa* Linn. was chosen to be verified of its anti-tumor effects in colon cancer using PDX models. One of the major components of turmeric, curcumin, has been shown to be anti-tumor in various cancer types in preclinical studies (39, 40). However, low water solubility, poor bioavailability, rapid metabolism, and systemic elimination of curcumin hinder its pharmacological application (41). Nevertheless, our previous studies demonstrated that turmeric ethanolic extract, which contains curcuminoids, turmerones, and other components, exerted potent anti-tumor and anti-metastatic effects as well as augmented anti-tumor activities of bevacizumab in colon cancer xenograft-bearing mice (19, 27, 42). Despite the mice models we used which covered both immuno-deficient nude mice and immuno-competent Balb/c mice, the xenografts were formed by homogeneous cell lines. Hence, the efficacy of TE treatment in colon cancer has been further verified using the PDX model in the present study, which would reveal the actual potential responders toward the treatments.

The present study demonstrated the anti-tumor and anti-metastatic effects of TE in six PDX samples. The responsive rate of anti-tumor effect toward TE was 50.0% (significantly reduced tumor weight in three out of six samples), and that of anti-metastatic effect was 83.3% responsive rate (five out of six) in both lungs and livers. Although the direct anti-tumor efficacy of TE was not as potent as that observed in cell-derived tumor-bearing model, the present findings further confirmed the potent anti-metastatic activities of TE treatment in PDX models, in which colon cancer cells from different patients metastasized to distal organs from the original xenografts. A recent study reported the inhibition of curcumin on tumor growth and aggressiveness using xenograft model with patient-derived oral squamous cell carcinoma cells (43). Nonetheless, the inhibitory effects of TE treatment toward lung and liver metastasis of colon PDX have not yet been reported. Interestingly, in our project, we did see all of moderately differentiated CRC tumors developed metastasis in PDX model. In fact, in clinical situation, around 70% of CRC tumor are moderately differentiated (44). For the hypothesis that moderately differentiated CRC tumors result in liver and lung metastasis, in our PDX model, we inoculated primary tumors into SCID mice, in which the immune surveillance to tumor metastasis is lacking. Meanwhile, not all primary tumors from patients could develop into PDX. Hence, the successful established PDX needs to be aggressive and may have potential to develop metastasis in patients as well. So, it is understandable for all models here turned out to be metastatic.

The samples of PDX after treatments were subjected to protein and molecular analysis in order to determine the mechanisms of action. Wnt signaling pathway controls cell fate, regulates homeostasis and is closely associated with malignant transformation (45). β-catenin is a key element in Wnt pathway and the loss of degradation of β-catenin in cytoplasm results in the transcription of cell proliferative, survival genes such as MYC, cyclin D (46). DKK1 encodes a Wnt suppressor gene which exerts pro-apoptotic and strong anti-proliferative activities in CRC cell lines (47, 48). TE treatment significantly up-regulated DKK1 expression in CC15OT sample (**Figure 5B**) and CC16ANT (data not shown) which might account for its anti-tumor effects. AXIN2 encoding a Wnt signaling component and promotes colon carcinoma oncogenic activity (49, 50). SMAD7 expression can affect β-catenin levels and lead to increased Wnt signaling (51). Results from IHC, Western blot and q-PCR showed the down-regulating effects of TE on Wnt signaling pathway, which involves in the CRC progression, including tumor initiation, growth and metastasis (52). In addition, KRAS and EGFR gene expressions were down-regulated by TE treatment in all tested PDX samples. EGFR signal pathway involves in angiogenesis, cancer cell proliferation, migration, invasion as well as inhibition to apoptosis (53). Furthermore, protein expression of Src was also down-regulated after TE treatment, indicating that TE also exerted anti-tumor and anti-metastatic effects through influencing cell growth, adhesion and migration (54). Vimentin, EpCAM, and N-cadherin (belong to EMT pathway) were all down-regulated in TE treatment group in tested PDX samples. These alternations of gene or protein expressions strongly suggested the multiple targets of TE treatment in colon cancer, such targets are also consistent with other studies regarding curcumin (55, 56).

On the other hand, the potential of TE treatment on inhibiting tumor recurrence in colon PDX model was firstly revealed in this study. Although the direct anti-tumor effect of TE treatment may not be as good as the first-line chemotherapeutics, the results of reduced numbers and sizes of recurrent tumors shown here should provide evidences on the “chemopreventive” efficacy of TE treatment. The molecular alterations after TE treatment may also explain the inhibition of tumor recurrence observed in the present PDX model. The results from the present study are of clinical significance as PDX model mimics clinical situation and predict the tumor metastasis. To the best of our knowledge, this is possibly the first study of herbal extract on PDX model of colon cancer.

The first-line chemotherapy for CRC in clinics is based on fluorouracil (5-FU) alone or in combination as FOLFOX (5-FU, folinic acid and oxaliplatin)/FOLFIRI (5-FU, folinic acid and irinotecan) for long time (35, 57). Unfortunately, despite effectiveness of the chemotherapeutics, the incidence of side effects is as high as 98% (58), including neurotoxicity, immune system suppression, hair loss, nausea and vomiting, severely affected the quality of life of patients (59). In the present study, two batches of mice bearing PDX samples were used to evaluate the efficacy of FOLFOX, which has seldom been reported. Similar to the side effects observed in patients, the body weight of mice bearing PDX received human equivalent dosages of FOLFOX decreased gradually (**Figure 7**). Meanwhile, the tumor growth was suppressed by FOLFOX treatment. The termination of treatment resulted in tumor volume increases. The overall outcomes of the incomplete FOLFOX treatment were not that positive, although the apoptotic activities retained (shown by TUNEL^+^ stained cells in **Figure 7C**). FOLFOX treatment did not effectively suppress liver metastasis in the mice-bearing PDX (**Figures 7C, F** **)** and it did not affect the Ki 67 and β-catenin expressions in tumor sections (data not shown). Taken together, the observed differential responses toward TE and FOLFOX treatments were as expected because the study subjects (i.e., each batch of PDX samples) were heterogeneous. In general, TE treatment might exert superior anti-metastasis efficacy than FOLFOX treatment in the PDX samples tested in this study.

Beyond the molecular mechanisms examined here, we utilized system biology method especially network pharmacology approach to predict the potential targets of TE based on its main components. Collectively, we constructed the protein-protein network of the 18 candidate targets of TE treated CRC in which the hub genes were revealed: AKT1, ESR1, and PTGS2. Furthermore, we discovered that the candidate targets may involve in signal transduction, inflammatory response, and positive regulation of nitric oxide biosynthetic process. Moreover, as the result of KEGG analysis, multiple pathways like PI3K-Akt signaling pathway and thyroid hormone signaling pathway, may be the potential pathways of turmeric in the treatment of CRC. We postulated that patients may benefit from the combination use of PI3K-Akt and/or thyroid hormone signaling pathway inhibitors together with TE treatment.

In conclusion, we have successfully established PDX model from a panel of CRC patients’ tumors which was a good platform for evaluating the multi-targeted herbal medicines. The preclinical anti-tumor and anti-metastatic activities of turmeric ethanolic extract were firstly demonstrated in individual colon PDX. To make this study more informative, we utilized network pharmacology to figure out more potential drug targets and working mechanisms. This precision medicine approach can potentially provide crucial information regarding preclinical translational evidence to clinical trials and together with network pharmacology to illustrate the potential therapeutic values of TE in CRC prevention and treatment.

## Data Availability Statement

The raw data supporting the conclusions of this article will be made available by the authors, without undue reservation.

## Ethics Statement

The studies involving human participants were reviewed and approved by Joint Chinese University of Hong Kong–New Territories East Cluster Clinical Research Ethics Committee. The patients/participants provided their written informed consent to participate in this study. The animal study was reviewed and approved by Animal Experimentation Ethics Committee of the Chinese University of Hong Kong. Written informed consent was obtained from the individual(s) for the publication of any potentially identifiable images or data included in this article.

## Author Contributions

CL and GY conceived the study. ML and GY performed the *in vitro* and *in vitro* studies and analyzed the data. LL performed the network pharmacology analysis. SN provided colon cancer patients’ tissue samples. ST, SN, and K-PF provided advices on study design and data interpretation. CL and K-PF contributed essential reagents and tools, ML, GY, and CL wrote the manuscript. All authors contributed to the article and approved the submitted version.

## Funding

This study was partly supported by grants of the State Key Laboratory of Research on Bioactivities and Clinical Applications of Medicinal Plants (CUHK) from Innovation and Technology Commission, HKSAR and the Vice-Chancellor’s One-off Discretionary Fund from the Chinese University of Hong Kong. This study was also partially supported by Si Yuan Foundation, Hong Kong.

## Conflict of Interest

The authors declare that the research was conducted in the absence of any commercial or financial relationships that could be construed as a potential conflict of interest.
