# Supplementary material for: Turmeric Is Therapeutic in Vivo on Patient-Derived Colorectal Cancer Xenografts: Inhibition of Growth, Metastasis, and Tumor Recurrence
Source: Front Oncol. 2021 Jan 19;10:574827. doi: 10.3389/fonc.2020.574827 (PMC7856407; doi:10.3389/fonc.2020.574827)
Supplement: Supplementary file 1 [file DataSheet_1.docx]

**Turmeric is therapeutic *in vivo* on patient-derived colorectal cancer xenografts: inhibition of growth, metastasis and tumour recurrence**

**Mingyue Li, Grace Gar-Lee Yue, Lianxiang Luo, Stephen Kwok-Wing Tsui, Kwok-Pui Fung, Simon Siu-Man Ng, Clara Bik-San Lau**

**Supplement information**

**Table S1**. Gene specific PCR primers

| **Genes** | **Forward Primer** | **Reverse Primer** |
| --- | --- | --- |
| **ABCA13** | AGGAGTGTGAGGCTCTTTGC | GGTGCTGTCCCTTGAACTGA |
| **ABCB1** | CTGCTTGATGGCAAAGAAATAAAG | GGCTGTTGTCTCCATAGGCAAT |
| **ASNS** | CACTCCGCGACTCCCTTTT | ACCATTTCCACGGATGCAA |
| **AXIN2** | ATTCGGCCACTGTTCAGACG | GACAACCAACTCACTGGCCTG |
| **CD9** | TGCATCTGTATCCAGCGCCA | CTCAGGGATGTAAGCTGACT |
| **DKK1** | AGCGTTGTTACTGTGGAGAAG | GTGTGAAGCCTAGAAGAATTACTG |
| **E-cadherin** | TTGACGCCGAGAGCTACAC | GTCGACCGGTGCAATCTT |
| **EpCAM** | AGTTGGTGCACAAAATACTGTCAT | TCCCAAGTTTTGAGCCATTC |
| **EGFR** | TCCCTCAGCCACCCATATGTAC | GTCTCGGGCCATTTTGGAGAATTC |
| **GAPDH** | CGAGATCCCTCCAAAATCAA | GGTGCTAAGCAGTTGGTGGT |
| **N-cadherin** | TCGCCATCCAGACCGACCCA | TGAGGCGGGTGCTGAATTCCC |
| **PDGFRB** | AGACACGGGAGAATACTTTTGC | AGTTCCTCGGCATCATTAGGG |
| **RTKN2** | ATGCTCGACTAATGGCCTATACA | CGTCGTGATCGTTCTTTATTGCT |
| **SMAD7** | GACAGCTCAATTCGGACAAC | TCTCGTAGTCGAAAGCCTTG |
| **Snail** | ATGCCGCGCTCTTTCCTCGTC | AGCAGGTGGGCCTGGTCGTAG |
| **vimentin** | ATTCCACTTTGCGTTCAAGG | CTTCAGAGAGAGGAAGCCGA |

**Table S2**. 14 potential active compounds of turmeric extract

| **Chemical Component** | **Pubchem Cid** | **2D Structure** |
| --- | --- | --- |
| Bisdemethoxycurcumin | CID: 5315472 | 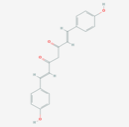 |
| 1,7-Bis(4-Hydroxy-3-Methoxyphenyl)-1,4,6-Heptatrien-3-One | CID: 10904292 | 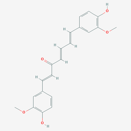 |
| 1,5-Bis(4-Hydroxy-3-Methoxyphenyl)-1,4-Pentadien-3-One | CID: 468133 | 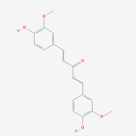 |
| 1,7-Bis(4-Hydroxyphenyl)-1,4,6-Heptatrien-3-One | CID: 71346280 | 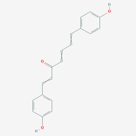 |
| Calebin A | CID: 637429 | 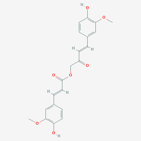 |
| Curcumin | CID: 969516 | 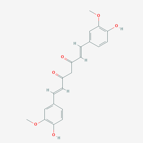 |
| Demethoxycurcumin | CID: 5469424 | 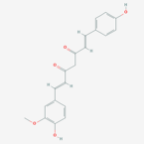 |
| Isoprocurcumenol | CID: 14543198 | 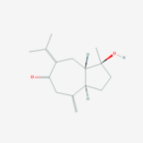 |
| Procurcumadiol | CID: 14633011 | 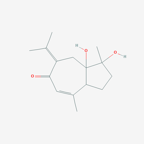 |
| Zedoarondiol | CID: 24834047 | 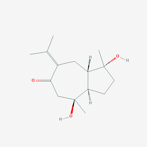 |
| Stigmasterol | CID: 5280794 | 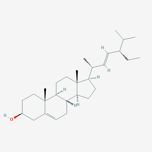 |
| campesterol | CID: 173183 | 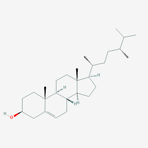 |
| cholesterol | CID: 5997 | 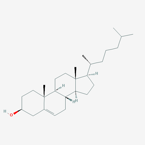 |
| 1Îo-Hydroxy-1,7-Bis(4-Hydroxy-3-Methoxyphenyl)-6-Heptene-3,5-Dione | CID: 68548065 | 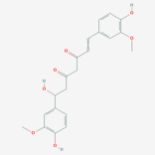 |

**
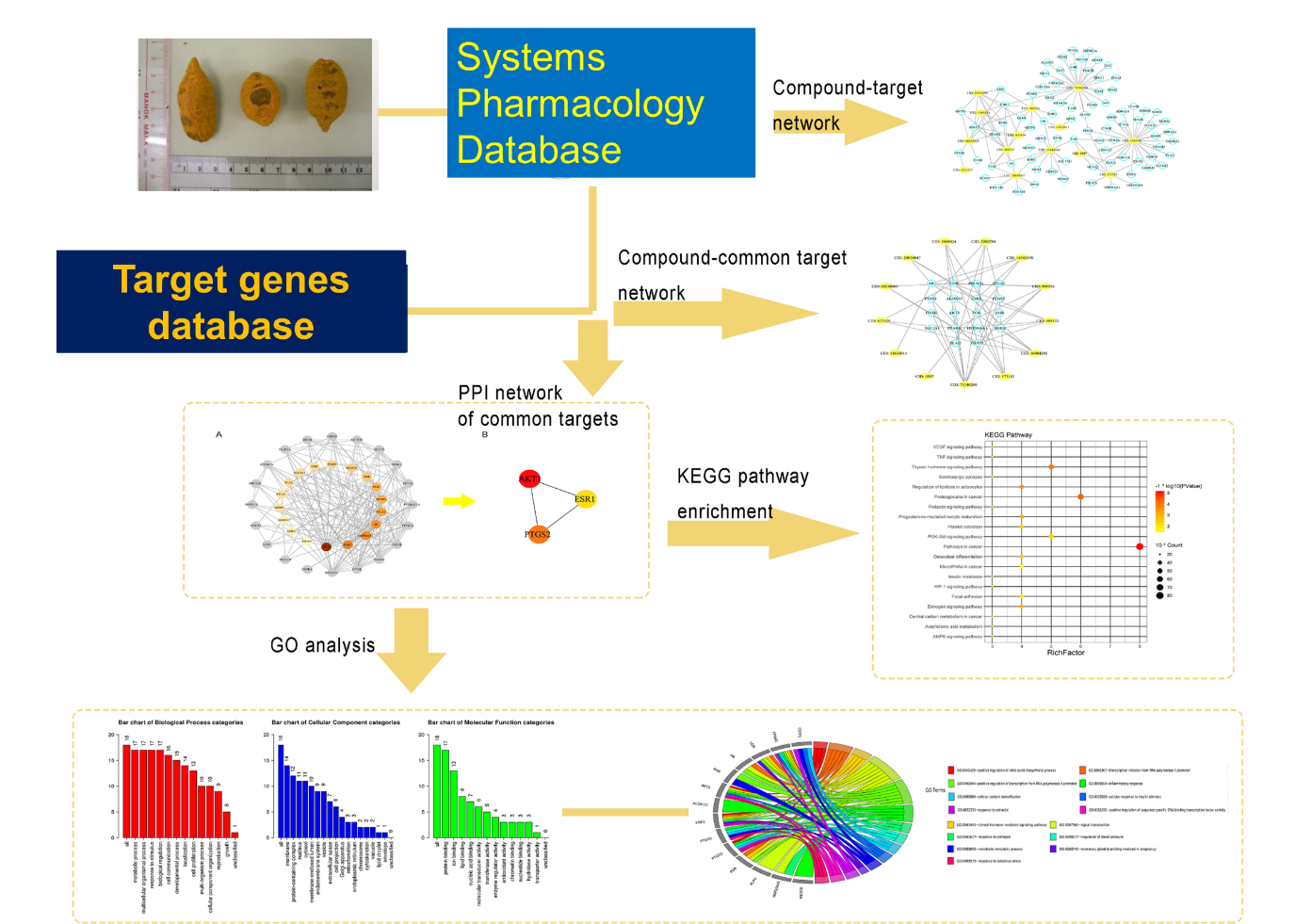
**

**Fig. S1 Network pharmacology workflow**


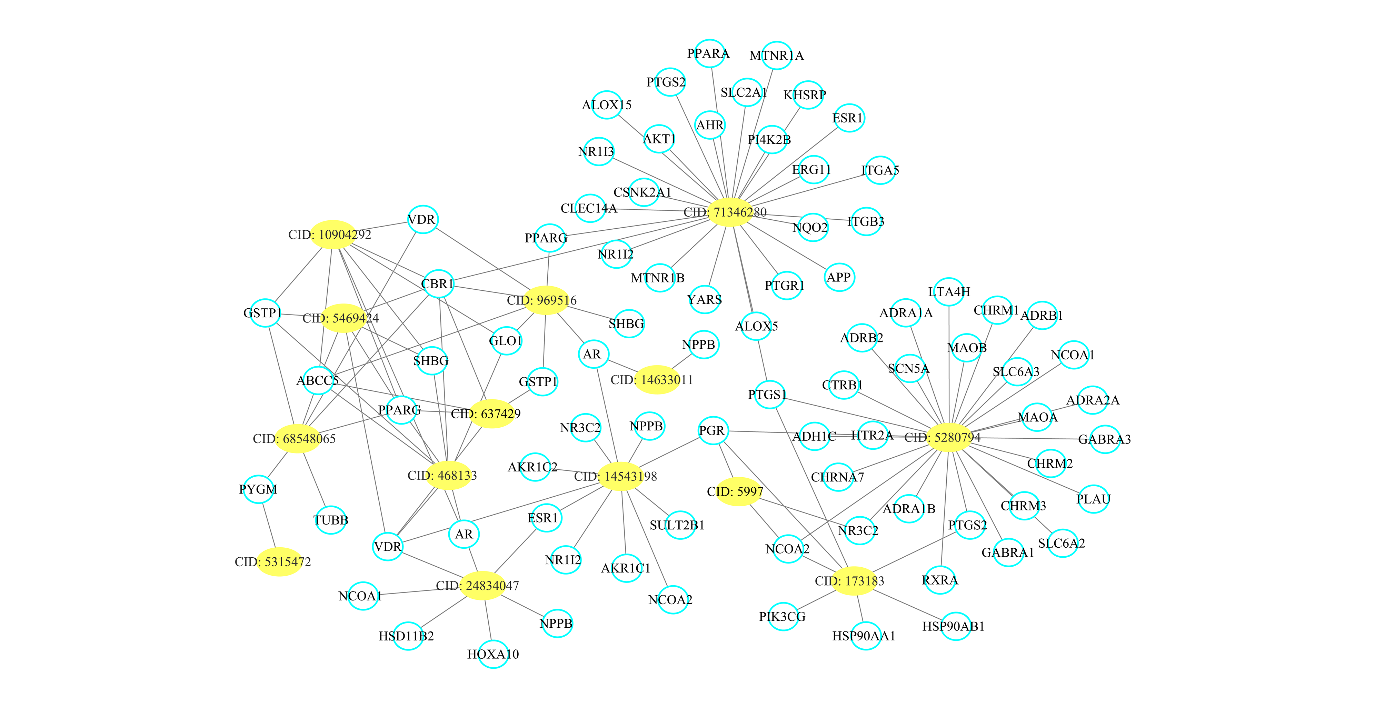


**Fig. S2 Compound-target Network of potential targets in Turmeric extract.** Yellow nodes stand for bioactive compounds from Turmeric extract. White nodes stand for putative targets.


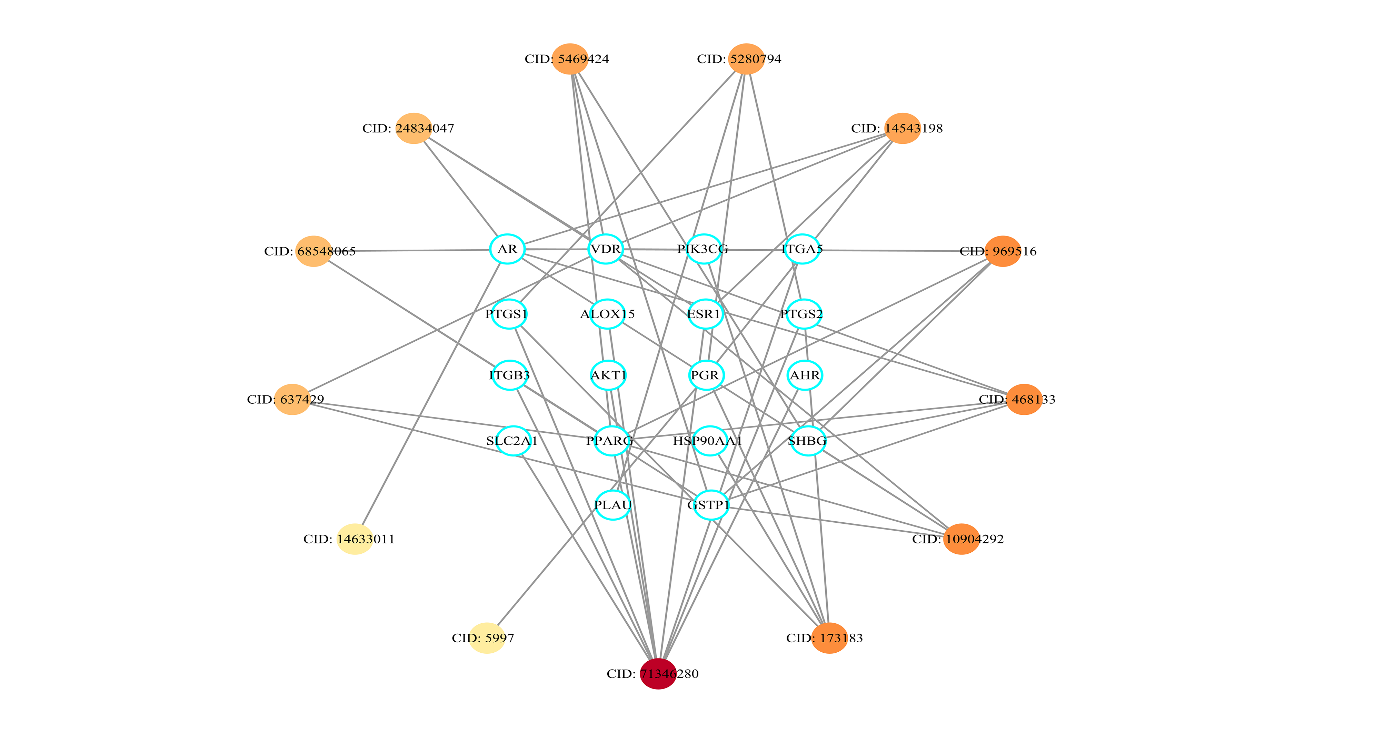


**Fig. S3 Compound-candidate target Network.** The white nodes represent candidate targets of Turmeric extract treated colorectal cancer. The colored nodes represent bioactive compounds from Turmeric extract. The darker the color, the greater the degree.


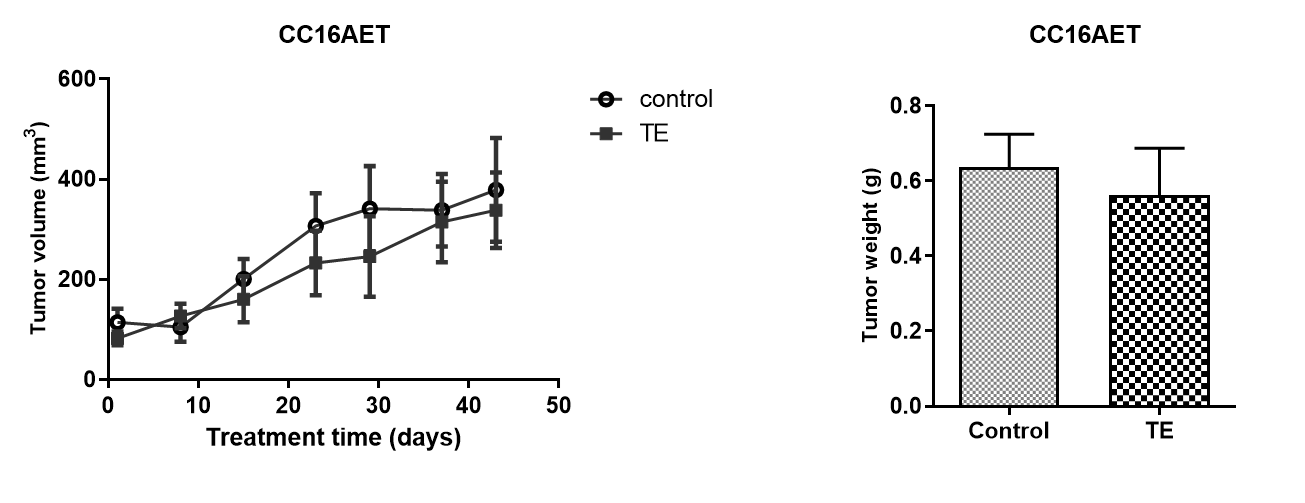


**Fig. S4** **Effects of TE treatment in PDX sample CC16AET.** The (A) tumor volume and (B) weight in the control group and TE treatment groups were shown (n = 7-8). Tumor volume was measured once a week while the final tumor weight was measured at the end of the experiment. Data were presented as mean ± SEM, no significant difference of the tumor weight between two groups.
